# MiR-126 and miR-146a as Melatonin-Responsive Biomarkers for Neonatal Brain Ischemia

**DOI:** 10.1007/s12031-023-02155-6

**Published:** 2023-09-19

**Authors:** Maria Cristina Albertini, Tania Vanzolini, Serafina Perrone, Michael D. Weiss, Giuseppe Buonocore, Valentina Dell’Orto, Walter Balduini, Silvia Carloni

**Affiliations:** 1 Department of Biomolecular Sciences, University of Urbino Carlo Bo, Via Saffi 2, 61029 PU Urbino, Italy; 2 Neonatology Unit, University Medical Center of Parma (AOUP) and University of Parma, Parma, Italy; 3 Department of Pediatrics, University of Florida, Gainesville, FL USA; 4 Department of Molecular and Developmental Medicine, University of Siena, Siena, Italy

**Keywords:** Hypoxic-ischemic encephalopathy, Biomarker, Melatonin, miRNA, Neonatal brain injury, Bioinformatics

## Abstract

Despite advances in obstetric and neonatal care, challenges remain in early identification of neonates with encephalopathy due to hypoxia-ischemia who are undergoing therapeutic hypothermia. Therefore, there is a deep search for biomarkers that can identify brain injury. The aims of this study were to investigate the serum and brain expressions of two potential biomarkers, miR-126/miR-146a, in a preclinical model of hypoxia-ischemia (HI)–induced brain injury, and to explore their modulation during melatonin treatment. Seven-day-old rats were subjected to permanent ligation of the right carotid artery followed by 2.5 h hypoxia (HI). Melatonin (15 mg/kg) was administered 5 min after HI. Serum and brain samples were collected 1, 6 and 24 h after HI. Results show that HI caused a significant increase in the circulating levels of both miR-126 and miR-146a during the early phase of ischemic brain damage development (i.e. 1 h), with a parallel and opposite pattern in the ischemic cerebral cortex. These effects are not observed 24 h later. Treatment with melatonin restored the HI-induced effects on miR-126/miR-146a expressions, both in the cerebral cortex and in serum. We conclude that miR-126/miR-146a are promising biomarkers of HI injury and demonstrate an associated change in concentration following melatonin treatment.

## Introduction

Neonatal encephalopathy (NE) due to hypoxia-ischemia (HI) is a severe pathological condition occurring in the brain (Descloux et al. 2015). HI triggers a sequence of pathophysiologic events that can lead to mortality and morbidity resulting in long-term neurologic deficits (Buonocore et al. 2022). HI leads to primary energy failure resulting in a decrease of ATP production and systemic acidosis (Berger and Garnier 1999). In turn, the primary energy failure causes a loss of the integrity of the neuronal cell membrane followed by a disequilibrium in calcium concentrations and subsequent cell death (Berger and Garnier 1999; Nair and Kumar 2018). The primary energy failure is followed by a secondary energy failure within 6–48 h after the hypoxic-ischemic insult. Secondary energy failure is characterized by high oxidative stress, inflammation, excitotoxicity and cell death. In a study involving infants, long-term or tertiary damage related to myelin deficits, reduced plasticity and altered cell number has been recorded months and years from the initial insult (Nair and Kumar 2018).

After the primary energy failure, a period of latency exists and therapeutic interventions during the latency phase of injury could possibly limit the neuronal damage (Nair and Kumar 2018). In infants suffering from moderate to severe NE, the only and standard treatment available is therapeutic hypothermia that seems to exert its neuroprotective effect by decreasing brain metabolism (Bustelo et al. 2020). Despite the therapy, several limitations have been recorded such as the narrow window of intervention, the need of a specialized and multidisciplinary clinical team and low percentage of treated neonates with normal long-term neurologic outcomes (Nair and Kumar 2018; Graham et al. 2016). Therefore, ongoing research is underway to develop adjunct therapies to be used in combination with therapeutic hypothermia to achieve improved long-term neurologic outcomes (Nair and Kumar 2018). Melatonin is one such adjunct therapeutic option that may augment the neuroprotective effects of hypothermia (Carloni et al. 2018). Melatonin is an endogenous indoleamine produced by the pineal gland and well known for its role in the regulation of the circadian rhythm, but it has also demonstrated to have significant functions in visual, reproductive, cerebrovascular, neuroendocrine and neuroimmunological systems (Carloni et al. 2017). Melatonin reduces the oxidative stress–related pathophysiologic states in newborns (Fulia et al. 2001); it crosses all morphophysiological barriers; moreover, its use did not result in any observable side effects (Buscemi et al. 2006; Gitto et al. 2013); hence, melatonin has emerged as a potential therapeutic agent for various neurological disorders and, among them, perinatal HI and inflammatory brain injuries (Chen et al. 2012). However, the proper timing of melatonin administration and the dose following injury in neonates are unknown. Studies have demonstrated that the effective therapeutic concentration of melatonin is higher compared to the physiological concentration (Balduini et al. 2019). Furthermore, particularly after neonatal brain injury, the neural damage may affect the processes related to melatonin secretion making its quantity even lower compared to a healthy subject (Fulia et al. 2001; Carloni et al. 2017). To promptly start the therapy, rapid and efficient diagnosis and prognosis are fundamental. The assessment of hypoxic-ischemic injury relies mainly on the clinical manifestations and on neuroimaging (cerebral ultrasound (CUS) and magnetic resonance imaging (MRI)) (Graham et al. 2016; Bersani et al. 2020). Despite the improvements in this field, identifying NE remains difficult due to the varied clinical presentations with lack of known timing of the original insult. Therefore, intervening in the early phases of injury, which could maximize the therapeutic benefits of melatonin, becomes nearly impossible (Nair and Kumar 2018). Thus, research focusing on biomarkers for detecting early injury to assist the bedside clinicians with objective information about the severity of injury is a high priority in perinatal medicine (Graham et al. 2016; Cho et al. 2019). The optimal biomarkers should be reliable, reproducible, safe, detectable, easy to detect, specific, sensitive, rapid and inexpensive. To date, only a few biomarkers meet these criteria and none of them is currently included in the standard monitoring procedures following hypoxic-ischemic injury in neonates (Bersani et al. 2020).

In this study, we aim to identify potential early biomarkers of brain injury following hypoxic-ischemic injury by examining microRNAs (miRNAs). MiRNAs are short (~20 nucleotides), single-stranded, endogenous, non-coding RNAs able to modulate the protein expression at a transcriptional and posttranscriptional levels (Cho et al. 2019; Ma and Zhang 2015; Peng et al. 2014; O’Brien et al. 2018). The central nervous system (CNS) is the main site of miRNAs expression with ~70% of those detectable findable in the brain and regulating CNS development and homeostasis (Carloni et al. 2017; Bersani et al. 2020). Recent studies have revealed the secretion of stable miRNAs also in serum and plasma of peripheral blood, making them intriguing biomarker candidates (Peng et al. 2014; Mens et al. 2021). Using a bioinformatics analysis, we found two different quantifiable miRNAs biomarkers, miR-126 and miR-146a, that could help in determining the ongoing brain injury and assist in bedside clinical management of neonatal patients. Indeed, we show that miR-126 and miR-146a, which are involved in either angiogenesis or inflammation, are altered in both the cerebral cortex and serum of pup rats exposed to HI. In addition, their modulation after melatonin treatment, which was reported to be neuroprotective in this model of HI-induced brain injury (Carloni et al. 2008), indicates the suitability of these miRNAs as new potential biomarkers for HI diagnosis.

## Materials and Methods

### Rat Cerebral Hypoxic-Ischemia

All animal procedures were performed in accordance with the Italian regulation for the care and use of laboratory animals (EU Directive 63/2010; Italian D.L. 26/14) and were approved by the Animal Care Committee of the University of Urbino Carlo Bo.

Pregnant Sprague-Dawley rats were housed in individual cages and the day of delivery was considered day 0. Neonatal rats from different litters were randomized, normalized to ten pups per litter and kept in a regular light/dark cycle (lights on 8 am–8 pm). On postnatal day 7, after anaesthesia with 3% isoflurane, pup rats underwent unilateral ligation of the right common carotid artery via a midline neck incision. After artery ligation, the wound was sutured, and the animals allowed to recover for 3 h under a heating lamp. Pups were then placed in an airtight jar and exposed for 2.5 h to a humidified nitrogen–oxygen mixture (92% and 8%, respectively) delivered at 5–6 L/min (HI). The jar was partially submerged in a 37 °C water bath to maintain a constant thermal environment (Carloni and Balduini 2008).

### Melatonin Treatment

Melatonin (Sigma-Aldrich, Milan, Italy, M5250) was dissolved in dimethyl sulfoxide (DMSO; Sigma-Aldrich, Milan, Italy, D5879) and diluted in normal saline solution to a final concentration of 5% DMSO (vehicle). The melatonin solution was intraperitoneally injected to pup rats 5 min after HI at the dose of 15 mg/kg (HI+Mel, *N*=18). Sham-operated controls (CTRL, *N*=18) and HI-injured animals (HI, *N*=18) received an equal volume of the vehicle. The melatonin dose was chosen based on previous experiments that showed protective effects of melatonin in the neonatal model of HI used in this study (Carloni et al. 2008). Animals were sacrificed at 1, 6 and 24 h after HI and melatonin treatment.

### Bioinformatics Analysis

Web‐based miRNet tool (https://www.mirnet.ca) was used to predict microRNAs modulation associated to the genes previously identified in a new NCHI (Neonatal Cerebral Hypoxic-Ischemia) pathway melatonin-sensitive (Weiss et al. 2022). The NCHI pathway genes list inserted in the analysis was the following: Wnt5, Frizzled, PTEN, PIP3, PI3K, sbac, Grb2, Sos, Ras, PKCa, Raf, MEK, ERK, Rb, p16, CDK4, Bak, p14, p21, Bax and p53. This application allowed us to create a network with the integration of multiple data types: NCHI pathway–related genes and neonatal HI brain injury. In the network, nodes were sorted by their degree/betweenness values and ordered depending on their importance to predict the most associated microRNAs. The hypergeometric distribution was selected to measure the statistical significance (Pval) of those microRNAs (miR-126 and miR-146a) identified from the genes list analysis (Chang et al. 2020).

### Quantitative Real-Time PCR for Mature miRNA Analysis

Pups were anesthetized and euthanized by decapitation 1, 6 or 24 h after HI and melatonin treatment. Blood samples were collected, stored overnight at room temperature and then centrifuged for 10 min at 2500 rpm and the serum collected. Brains was rapidly removed and the homogenate supernatants from cerebral cortices were prepared as previously described (Carloni et al. 2016). Briefly, the cerebral cortices were sonicated in 0.4-mL lysis buffer containing 1M Tris, 0.25M EDTA, 0.025M EGTA, 10mM phenylmethylsulfonyl fluoride (PMSF) in absolute EtOH and f-complete protease inhibitor cocktail, using an Ultrasonic Liquid Processor XL Sonicator. Homogenates were centrifuged for 10 min at 15,000 rpm (4 °C) and the supernatants aspirated and stored at −20 °C until the RNA extraction.

The miRNAs were isolated from the serum and cortical homogenate supernatants using the Norgen total RNA isolation kit (Weiss et al. 2022). Rat miR-126 and miR-146a (brain U6 and serum spike-in cel-miR-39 reference miRNAs) expressions were evaluated using the TaqMan miRNA assay. The TaqMan miRNA reverse transcription kit was used to reverse transcribe miRNAs. Subsequently, RT-qPCR was performed in 20 μL of PCR mix containing 1 μL of 20× TaqMan miRNA assay, which contained PCR primers and probes (5′-FAM), 10 μL of 2×TaqMan Universal PCR Master Mix No Amp Erase UNG and 5 μL of reverse-transcribed product. The reaction was first incubated at 95 °C for 10 min followed by 40 cycles at 95 °C for 15 s and at 60 °C for 1 min. The quantitative real-time PCR (RT-qPCR) was performed on a ABIPRISM 7500 Real Time PCR System. Data were analyzed by a 7500-system software (1 1.4.0) with the automatic comparative threshold (Ct) setting for adapting baseline. Detection thresholds were set at 35 Ct. The relative amounts of miR-146a and miR-126 were calculated using the Ct method: ΔCt = Ct (miR-146a/miR-126) − Ct (refence miRNA); 2ΔCt. Results are expressed in the figures as fold induction relative to control values (Carloni et al. 2017).

### Data Analyses

Statistical analyses were performed by two-way ANOVA or one-way ANOVA using the Prism Computer program (GraphPad Software Inc.). Bartlett’s test was used to determine data homogeneity. The Bonferroni multiple comparison test or Newman–Keuls multiple comparison test was used to determine differences between groups. Results were considered to be significant when *p* ≤0.05.

## Results

### Predicted MicroRNAs Modulated in the NCHI Pathway Melatonin-Sensitive

**Fig. 1:**
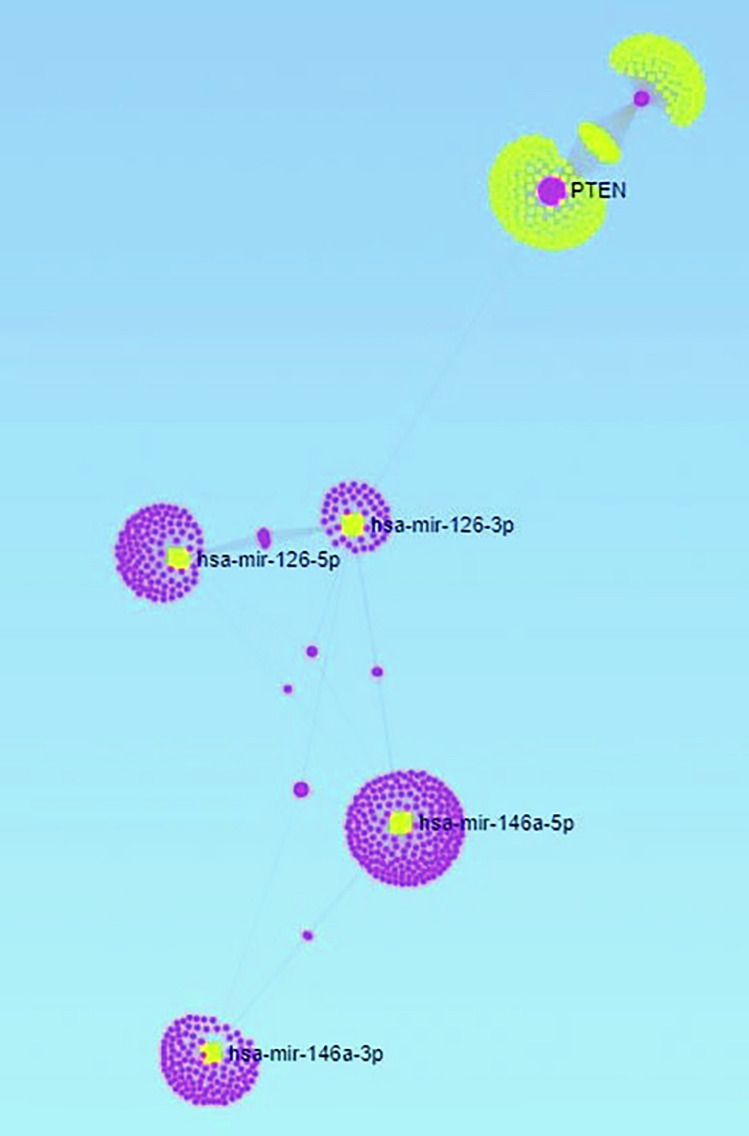
Network created by the miRNet bioinformatics analysis of NHCI pathway genes and hypoxic-ischemic encephalopathy. The miR-126 and miR-146a families were the most statistically significant miRNAs related to the network identified. The miRNAs are indicated in yellow while the target genes are indicated in pink. The nodes indicate the most significant interactions between miRNAs (yellow node) and their target genes (pink node)

We have previously identified a new NCHI pathway that is melatonin-sensitive with specific genes involved in the pathway (Weiss et al. 2022). We decided to exploit this pathway through miRNet bioinformatics tool to perform a mixed input analysis (multiple query type) with NHCI pathway genes and hypoxic-ischemic encephalopathy. As indicated in Fig. 1, the network obtained by the analysis predicted that the most important associated miRNAs nodes (highest degree/betweenness values) were related to miR-126 and miR-146a families (Pval = 0.0000063 and 0.0000314 respectively).

### MiR-126 and miR-146a are Modulated After Neonatal Brain Ischemia and During Melatonin Treatment

**Fig. 2:**
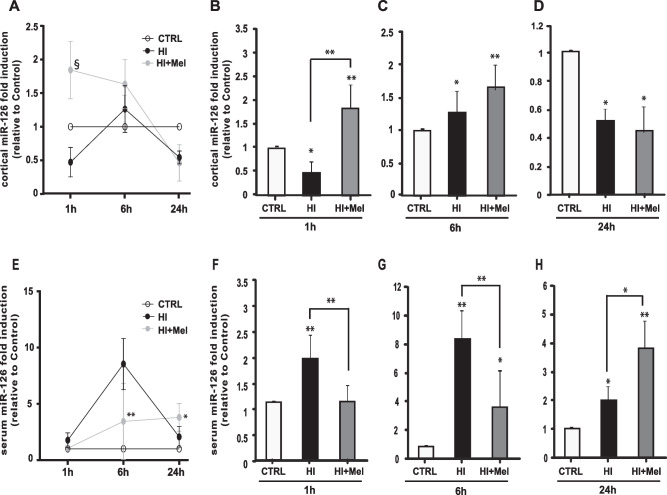
Expression of miR-126 in the cerebral cortex (**A**, **B**, **C**, **D**) and serum (**E**, **F**, **G**, **H**) of control (CTRL), hypoxic-ischemic (HI) and melatonin-treated hypoxic-ischemic (HI + Mel) neonatal rats at 1 h (**B**, **F**), 6 h (**C**, **G**) and 24 h (**D**, **H**) after the injury and melatonin treatment. Values are indicated as fold change related to CTRL rats. The results represent the mean value of 6 independent experiments (*n* = 6 animals/group). In **A** and **E**, * *p*≤0.05, ** *p*≤0.01, § *p*≤0.001 vs HI, two-way ANOVA followed by Bonferroni multiple comparison test. In **B**–**D** and **F**–**H**, * *p*≤0.05, ** *p*≤0.01 vs CTRL, * *p*≤0.05, ** *p*≤0.01 (lines), one-way ANOVA followed by Newman–Keuls multiple comparison test

**Fig. 3:**
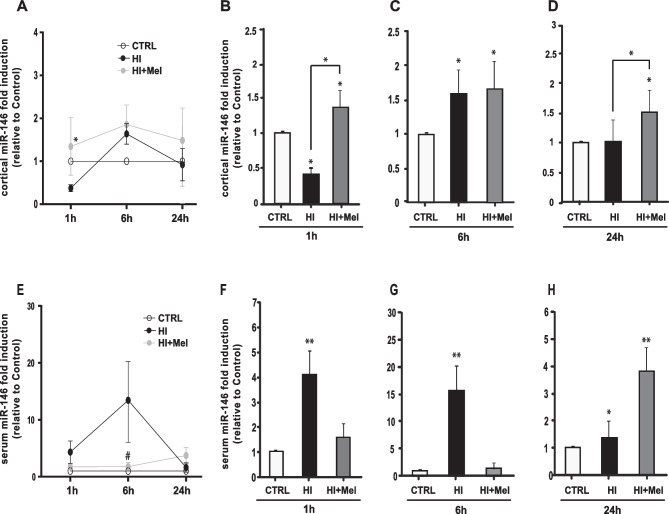
Expression of miR-146a in the cerebral cortex (**A**, **B**, **C**, **D**) and serum (**E**, **F**, **G**, **H**) of control (CTRL), hypoxic-ischemic (HI) and melatonin-treated hypoxic-ischemic (HI + Mel) neonatal rats at 1 h (**B**, **F**), 6 h (**C**, **G**) and 24 h (**D**, **H**) after the injury and melatonin treatment. Values are indicated as fold change related to CTRL rats. The results represent the mean value of 6 independent experiments (*n* = 6 animals/group). In **A** and **E**, * *p*≤0.05, # *p*≤0.01 vs HI, two-way ANOVA followed by Bonferroni multiple comparison test. In **B**–**D** and **F**–**H**, * *p* ≤ 0.05, ** *p* ≤ 0.01 vs CTRL, * *p*≤0.05 (lines), one-way ANOVA followed by Newman–Keuls multiple comparison test

To evaluate if the predicted miR-126 and miR-146a were modulated after HI and melatonin treatment, qRT-PCR analysis of miRNAs levels in both the cerebral cortex and serum of animals sacrificed 1, 6 and 24 h after HI and melatonin treatment was performed (Figs. 2 and 3). One hour after HI, compared to CTRL, miR-126 levels decreased in the cerebral cortex of HI animals (Fig 2A; *p*≤0.05, Fig. 2B), increased after 6 h (Fig 2A; *p*≤0.05, Fig. 2C) and decreased again 24 h after the injury (Fig 2A; *p*≤0.05, Fig. 2D). In HI animals treated with melatonin, instead, miR-126 cortical expression significantly increased at 1 h and 6 h after injury compared to CTRL (*p*≤0.01, Fig. 2B and p≤0.01, Fig. 2C, respectively) and at 1 h compared to HI animals (*p*≤0.001, Fig. 2A), whereas its expression was equivalent to that found in the cerebral cortex of HI animals 24 h later (Fig 2D). Evaluation of the miR-126 levels in the serum revealed an opposite pattern of circulating miR-126 compared to the cerebral cortex (Fig. 2E). Indeed, the serum miR-126 expression significantly increased after HI at all the time points analyzed compared to the levels measured in the serum of CTRL animals (*p*≤0.01, Fig. 2F and G; *p*≤0.05, Fig. 2H). On the contrary, melatonin significantly decreased miR-126 serum expression at both 1 h (*p*≤0.01, Fig. 2F) and 6 h after injury (*p*≤0.01, Fig. 2E and G) and increased it after 24 h compared to HI ischemic animals (*p*≤0.05, Fig. 2H).

A similar pattern was observed for the miR-146a expression (Fig. 3). Compared to the CTRL, miR-146a cortical expression was down-regulated 1 h after HI (*p*≤0.05, Fig. 3B), up-regulated after 6 h (*p*≤0.05, Fig. 3C) and returned to control levels after 24 h (Fig. 3D). In the cerebral cortex of HI animals treated with melatonin, miR-146a levels increased at all the time points analyzed compared to CTRL (*p*≤0.05, Fig. 3B–D) and at 1 h and 24 h after injury compared to HI group (*p*≤0.05, Fig. 3A, B and D). As shown in Fig. 3, panels E–H, HI significantly increased miR-146a serum expression during the early phase of ischemic brain damage development (*p*≤0.01, Fig. 3F and G; *p*≤0.01 Fig. 3H). Melatonin treatment, instead, maintained miR-146a serum levels similar to control values at both 1 h and 6 h after HI (Fig. 3F and G, respectively), whereas stimulated an increase over CTRL levels after 24 h (*p*≤0.01, Fig. 3H).

## Discussion

Neonatal mortality contributes to 46% of under-five mortality globally (UNICEF DATA [Internet] 2021). NE is responsible for a high number of these deaths (Lawn et al. 2005), which could be potentially avoided through adequate monitoring techniques and proper and rapid diagnoses. Current diagnostic criteria for NE due to HI in the early hours lack objective measurement tools and, although advances in the field of biomarkers are currently ongoing, delays and several bottlenecks are affecting the approval for their use as standard monitoring tools in the clinical care of neonates with potential hypoxic-ischemic brain injury. During critical clinical bedside decision periods in the care of neonates with hypoxic-ischemic brain injury, there is a lack of specific brain injury–related proteins. MiRNAs have raised more and more interest as brain-specific markers because, according to recent studies, they are not only primarily expressed in the brain but they are also secreted in plasma (Peng et al. 2014). Their detectability in peripheral blood allows miRNAs to be promising candidates for utilization in the clinical care of neonates with NE. Using the web‐based miRNet tool and creating a network with the integration of the data “NCHI pathway melatonin-sensitive” (Weiss et al. 2022) and “neonatal Hypoxic-Ischemic brain injury”, we identified miR-126 and miR146a as potential biomarkers that could be useful for diagnostic purposes in perinatal brain injury. To assess the suitability of these miRNAs, we utilized a neonatal rat model of HI brain injury that allows us to assess the release of miRNAs in serum and their concomitant expression in the brain. Among different miRNAs identified as potential biomarkers for the detection of the HI brain injury (Li et al. 2022), both miR-126 and miR146a were highlighted in previous investigations in adult models of stroke (Tan et al. 2021; Qi et al. 2020; Fullerton et al. 2022), but their expressions were not investigated after neonatal HI and melatonin treatment. Here, we observed opposite pattern between miR-126 and miR146a expression in the neonatal ischemic tissue and serum, firstly indicating that the increased circulating miRNAs levels are a direct consequence of the neonatal HI insult, and then suggesting the suitability of the use of these miRNAs as a source of information for HI appraisal (Weiss et al. 2022). Furthermore, miR-126 and miR146a dysregulations were observed within 1 h from the end of neonatal HI insult, which is inside the well-known latent period in which medical interventions, like hypothermia or melatonin, can produce the maximal beneficial effects (Yang et al. 2020). After melatonin, miRNA serum levels were found to be similar to those measured in the control and, at the same time, over-expressed in the cerebral cortex, supporting the correlation between circulating miRNAs and brain damage. This can suggest an activation of compensatory mechanisms of protection that lead peripherally to a control of the miRNAs release and, in the site of injury, to the enhancement of tissue repair processes. Indeed, although not directly assessed in this study, the dose and schedule of melatonin administration used have been repeatedly shown to be neuroprotective in this model of neonatal HI brain injury, reducing the long-term behavioural deficits as well as the brain damage induced by HI (Carloni et al. 2008; Alonso-Alconada et al. 2012). Accordingly, we previously observed that melatonin, at the same experimental conditions, not only significantly reduced the oxidative stress, the inflammation as well as the endoplasmic reticulum stress induced by neonatal brain ischemia (Carloni et al. 2014, 2016), but also early activated autophagy and rapidly rescued the HI-induced silent information regulator 1 (SIRT1) depletion (Carloni et al. 2017), both having have a significant role in the complex and dynamic signalling network involved in neuroprotection. Moreover, we previously found miRNAs modulation in serum of HI encephalopathy neonate after 12- and 48-h melatonin infusion, showing upregulation of miR-126 and miR-146a circulating levels (Weiss et al. 2022).

MiR-126 is the most expressed vascular miRNA in primary human endothelial cells (ECs) derived from veins, arteries, skin and brain, and seems to be involved in maintaining vascular integrity, in the regulation of endothelial adhesion molecules during inflammation and in angiogenesis (Cerutti et al. 2017). Deletion of miR-126 delayed vascular development in the retina and brain, impaired VEGF-dependent corneal angiogenesis in adults (Kuhnert et al. 2008), and affected ECs proliferation, migration and angiogenesis, resulting in the lack of vascular integrity, haemorrhaging and partial embryonic lethality (Ma and Zhang 2015). In our experiments, after neonatal HI brain injury, miR-126 levels in the cerebral cortex were down-regulated compared to the controls at 1 and 24 h. These results might be explained by the pathophysiologic evolution of HI-induced neonatal brain damage. As reviewed by Kleuskens and colleagues, the acute phase of HI injury is characterized by anaerobic metabolism arising within the first 30 min following the initial insult continuing over the next 6 h (Kleuskens et al. 2021). This acute phase of HI injury is characterized by inflammation, oxidative stress, excitotoxicity and cell death and, consequently, cellular response to blood reperfusion (Qin et al. 2022). Necrosis rapidly appears in ischemic brain areas and has an extended role in the progression of neonatal HI brain injury (Carloni et al. 2007). In this scenario, the amount of miR-126, reduced at 1 h and increased at 6 h during the acute phase of injury, may be considered consistent with the cellular damage and the subsequent attempt to restore the blood flux and reduce hypoxia by angiogenesis, respectively. Indeed, reperfusion may occur 2 to 5 h after the hypoxic-ischemic event, and can continue for several days (Kleuskens et al. 2021). At 24 h, miR-126 expression was reduced again, and this is a timeframe in which a plethora of signalling pathways, either detrimental or neuroprotective, are already activated and involved in the ischemic brain damage pathophysiology. A similar trend for miR-126 expression was described by Gai and colleagues who observed miR-126 reduction during the progression of the ischemic brain damage in adult rats affected by MCAO (Gai et al. 2019). In serum, miR-126 levels after HI insult were higher compared to the control at each time point studied. Melatonin administration after the HI injury was associated with an increase of miR-126 at 1 and 6 h in the cerebral cortex, confirming the potential neuroprotective effect of melatonin in ischemic tissue repair that we previously observed (Carloni et al. 2008, 2017). Twenty-four hours after HI, the potential effect of melatonin on increased miR-126 levels is no longer observed, probably due to the pharmacokinetic profile of melatonin. Considering the data collected from both animal and human studies, melatonin pharmacokinetic profile differs between neonates and adults (both humans and animals) (Carloni et al. 2017; Merchant et al. 2013); hence, it is possible that, 24 h after melatonin administration, another dose is needed to reach a steady therapeutic concentration (Balduini et al. 2019). Furthermore, the neuroprotective action of melatonin occurs at doses much higher than those used to restore the physiological values (ranging from 5 to 15 mg/kg) (Carloni et al. 2008). On the other hand, melatonin tends to restore the basal levels of miR-126 in serum, reducing the miRNA levels increased by HI in the acute phase of HI injury, whereas these melatonin effects are not observed after 24 h.

MiR-146a is a negative regulator of inflammation. It is usually modulated in presence of different pathological neurological conditions to compensate chronic inflammation and restore homeostasis (compensatory anti-inflammatory response) (Carloni et al. 2016; Gaudet et al. 2018). In the HI-injured cortex, 1 h after the injury, miR-146a was lower compared to the control, and increased after 6 h; these results are consistent with the pathophysiologic phase of acute inflammation between 6 and 15 h observed after the HI insult (Carloni et al. 2007; Jayaraj et al. 2019). Twenty-four hours after HI, miR-146a expression returned to the control concentrations, suggesting that the compensatory anti-inflammatory response, aimed to restore the cellular homeostasis and prevent the secondary energy failure, is activated early after the hypoxic-ischemic insult. These results are also in line with studies showing that pathways involved in the innate immunity such as the Toll-like receptor signalling, in which miR-146a seems to play a key role, are no longer activated in the late phase of ischemia-reperfusion injury (Chen et al. 2013). As for miR-126, miR-146a levels in serum of ischemic animals were higher compared to the control at each time point studied, revealing an unreflective pattern of miR-146a expression in circulation and cerebral cortex, especially in the early phase of ischemic brain damage development (i.e. 1 h after HI). These data support the possibility to use circulating miR-146a evaluation as a non-invasive predictive biomarker of neonatal hypoxic-ischemic brain injury as recently suggested for other several diseases (Shahid et al. 2021; Quan et al. 2018; Ballinas-Verdugo et al. 2021). Melatonin reduced the miR-146a release in circulation of neonatal HI rats, suggesting its positive effect on activation of the compensatory anti-inflammatory response. Indeed, melatonin has been shown to possess anti-inflammatory effects (Balduini et al. 2012) through several mechanisms of action among which miR-146 modulation was recently mentioned (Su et al. 2022; Zhou et al. 2022).

In summary, this study reported preclinical evidence of a significant dysregulation of miR-126 and miR-146a in neonatal rats in the early phase of HI injury and restored effects after melatonin treatment. Results highlight the opposite pattern between the miRNAs expression in tissue and serum, respectively, indicating the increased circulating miRNAs levels as a direct consequence of the HI insult. Furthermore, both circulating miR-126 and miR-146a are highly expressed within 6 h from HI. It is known that the therapeutic window of intervention leading to successful outcomes in human neonates with NE due to HI injury is estimated to be between 6 and 15 h until a few days after the insult (Vannucci and Perlman 1997). In light of this, the remarkable difference compared to basal level in such a short period of time is an exquisite advantage in terms of diagnosis but also a great opportunity to rapidly and efficiently start a therapy. Although we point out the limit of this study as a preclinical step, and that further explorations are needed to determine whether these conclusions hold true in the clinical setting, we consider these data as helpful to improve the panel of tools that can be used to determine the ongoing brain injury in neonatal patients and to assist in bedside clinical management.

## Data Availability

The datasets generated during and/or analyzed during the current study are available from the corresponding author on reasonable request.

## Abbreviation

HI: Hypoxia-Ischemia
NCHI: Neonatal Cerebral Hypoxic-Ischemia
NE: Neonatal Encephalopathy
